# Oral language profiles and associated factors in children after neonatal arterial ischaemic stroke

**DOI:** 10.1111/dmcn.70132

**Published:** 2025-12-30

**Authors:** Laure Drutel, Virginie Dardier, Lena Avoyan, Lucie Hertz‐Pannier, Mickaël Dinomais

**Affiliations:** ^1^ Laboratoire de Psychologie, Cognition, Comportement, Communication (LP3C) Rennes 2 University Rennes France; ^2^ French National Reference Center for Pediatric Stroke Les Capucins Pediatric Medical and Rehabilitation Care Angers France; ^3^ Centre Hospitalier Universitaire de Poitiers Poitiers France; ^4^ UMR 1141, NeuroDiderot, inDEV Team Paris Cité University, INSERM, AP‐HP Paris France; ^5^ UNIACT Neurospin, CEA‐Saclay Gif sur Yvette France; ^6^ Department of Physical Medicine and Rehabilitation University Hospital of Angers, Faculté de Santé Angers France; ^7^ Laboratoire Angevin de Recherche en Ingénierie des Systèmes (LARIS) University of Angers France

## Abstract

**Aim:**

To characterize language outcomes at age 7 years after neonatal arterial ischaemic stroke (NAIS) and identify language profiles and determinants.

**Method:**

This prospective longitudinal cohort study included 70 children (44 males) from a French cohort with NAIS. Oral language (phonology, lexicon, syntax) was assessed using a validated French battery. Data on demographics, environment, lesion, epilepsy, motor status, cognition, schooling, and therapy were collected. A data‐driven classification, based on principal component analysis, followed by hierarchical clustering with *k*‐means consolidation, was used to identify the language profiles. Performances were compared to norms and factors were tested using multiple linear regression models.

**Results:**

Four language profiles emerged after excluding three outliers: preserved language (*n* = 32), very low phono‐syntax (*n* = 7), very low receptive (*n* = 11), and borderline language (*n* = 17). A majority of children (56%) showed below‐age language abilities. Epilepsy, bilingualism, a family history of language or learning disorders, and lower full‐scale IQ were associated with poorer outcomes. Children with more severe impairments more often required school support (*n* = 18) or specialized education (*n* = 7). Access to speech and language therapy was mainly driven by the nature of difficulties, especially phonology and syntax, than by their severity.

**Interpretation:**

NAIS leads to frequent and heterogeneous language difficulties at school age, which are influenced by biological, cognitive, and environmental factors. Systematic and complete language assessments are crucial in preschool to ensure early detection, even of subtle deficits, and equitable access to intervention.

## Graphical abstract

**This original article is commented on by Westmacott on pages** 1026‐1027 **of this issue.**

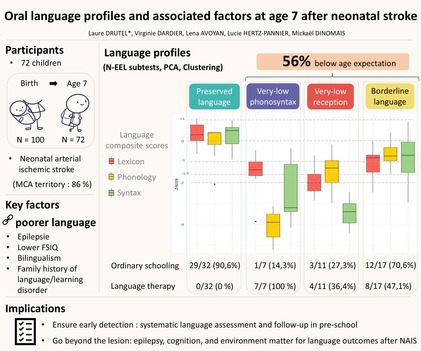

**Abbreviations**
FSIQ: full‐scale IQ
NAIS: neonatal arterial ischaemic stroke
SES: socioeconomic status

**What this paper adds**

- Four distinct language profiles emerged at age 7 years after neonatal arterial ischaemic stroke (NAIS).
- Over half of children with NAIS showed persistent language impairments at age 7 years.
- Epilepsy, full‐scale IQ, bilingualism, and a family history of language or learning disorders are key determinants.
- Receptive or subtle impairments are under‐referred for language intervention.

Language development is shaped by biological, cognitive, and socio‐environmental factors, which are closely linked to brain maturation and plasticity.^1^, ^2^, ^3^ Early brain injuries can significantly affect neurodevelopment, increasing the risk of motor, cognitive, and language impairments.^4^ Because language skills are essential for cognitive, affective, and social development,^5^ understanding long‐term language outcomes after neonatal arterial ischaemic stroke (NAIS) is crucial.

NAIS, the most common form of perinatal stroke, affects approximately 1 in 3000 term births.^6^ It occurs in the first 28 days of life, typically in the first week, and predominantly affects the left middle cerebral artery territory.^7^ This early focal injury provides a unique model to study developmental plasticity and its limits.

Despite its clinical relevance, the school‐age outcome after NAIS remains understudied.^8^ At this age, 60% of children exhibit neurodevelopmental disabilities, most often in mild forms.^9^ The study of a French cohort with NAIS provided a unique broad overview of outcomes at age 7 years, including global language abilities;^8^, ^10^ however, previous publications were restricted to limited language descriptive data.

This prospective longitudinal cohort study aimed to further characterize language outcomes at age 7 years in this cohort, examining its variability, determinants, and functional consequences. Given the complex interplay between neurological, biological, and socio‐environmental factors, a single, uniform language profile is unlikely to emerge after NAIS.^11^ The significant variability observed from the earliest stages of language development after NAIS further supports the expectation of varied language profiles in school‐age children.^12^ Therefore, we hypothesized that distinct oral language profiles would emerge. We also expected language outcomes to be shaped by lesion characteristics (e.g. laterality, vascular territory), epilepsy, and environmental factors, such as socioeconomic status (SES).^11^, ^13^, ^14^, ^15^, ^16^, ^17^ Furthermore, we anticipated that more severe language difficulties would be associated with increased use of educational and speech therapy support.

## METHOD

### Ethics statement

This study was conducted in accordance with international ethical standards and the Declaration of Helsinki. It was approved by the regional ethics committee in May 2010 (ClinicalTrials.gov registration: NCT02511249; Programme Hospitalier de Recherche Clinique Régional no. 0308052, no. 1008026, and EudraCT 2010‐A00329‐30). Written informed consent was obtained from each family.

### Participants and descriptive data

The study population met the following inclusion criteria: infants born at term with symptomatic NAIS confirmed using diffusion‐weighted magnetic resonance imaging before day 8 of life enrolled in the French cohort with NAIS (*n* = 100) and assessed at age 7 years between 2010 and 2013 through a clinical evaluation that included motor, cognitive, and language assessment (*n* = 72). The non‐inclusion criterion was severe global cognitive impairment preventing language testing (*n* = 2); this yielded a final sample of 70 participants. No participants had hearing impairments (for further details, see previous publications^6^, ^10^).

Demographic and clinical variables were collected, including age, sex, family background (SES, sibling rank, history of language or learning disorders, multilingualism), lesion characteristics (side, arterial territory, cortical or subcortical involvement), epilepsy, motor status, intellectual efficiency, schooling, and speech and language therapy (Table 1).

**TABLE 1:** Descriptive data of the NAIS: language group at age 7 years.

Characteristic	Overall (*n* = 70)
Age (year:month), median (Q1–Q3)	7:1 (7:0–7:2)
Sex	
Male	44 (62.9)
Birth order	
First	37 (52.9)
Socioeconomic status, mean (SD)	35.6 (13.0)
Exposure to bilingualism	12 (17.1)
Family history of language or learning disabilities (*n* = 69)	6 (8.6)
Epilepsy	15 (21.4)
Cerebral palsy	21 (30.0)
NAIS side	
Left	46 (65.7)
Right	19 (27.1)
Bilateral	5 (7.1)
Injury involving middle cerebral artery	60 (85.7)
NAIS location	
Superficial	48 (68.6)
Deep	8 (11.4)
Both	14 (20.0)
Full‐scale IQ (*n* = 65), mean (SD)	94.9 (19.1)
Perceptual Reasoning Index (*n* = 69), mean (SD)	93.4 (17.9)
Schooling (*n* = 70)	
Ordinary school	45 (64.3)
Ordinary school with support	20 (28.6)
Specialized school	5 (7.1)
Speech therapy	22 (31.4)

Data are shown as *n* (%) unless otherwise stated.

Abbreviations: NAIS, neonatal arterial ischaemic stroke; Q, quartile.

SES was determined using the Hollingshead Four‐Factor Index of Social Status score adapted for the French system, as described previously,^18^ and independently scored by two evaluators with discrepancies resolved by consensus. Higher scores indicate higher SES. Family history was based on parent‐reported language or learning disorders in siblings or parents. Epilepsy was defined as two or more unprovoked seizures, or a single seizure requiring prolonged antiseizure treatment, after the neonatal period.^10^ Motor status was defined as the presence or absence of unilateral cerebral palsy (CP) according to the Surveillance of Cerebral Palsy in Europe criteria. Global intellectual functioning was evaluated using the Wechsler Intelligence Scale for Children, Fourth Edition. Full‐scale IQ (FSIQ) was calculated only if index score differences were non‐significant. In addition, the Perceptual Reasoning Index, a non‐verbal reasoning measure excluding verbal subtests and minimizing motor speed demands, was also computed for supplementary analyses. The schooling context was recorded at the time of assessment: grade level, delays or advancements, and type of schooling. Three categories were defined: mainstream schooling without support; mainstream schooling with individualized support, such as an adult assisting the child in class, help from school‐based teams, or ongoing support from mobile special education and therapy services; and specialized schooling, including specialized units within mainstream schools and dedicated institutions for children with significant cognitive disabilities. Group characteristics were consistent with the literature, suggesting the representativeness of the broader population with NAIS.^7^, ^8^, ^9^ Among the 70 children, 12 grew up in a bilingual environment: French with Arabic (*n* = 5), English (*n* = 1), Turkish (*n* = 1), Portuguese (*n* = 2), Ivorian dialect (*n* = 1), Congolese dialect (*n* = 1), or French Creole (*n* = 1). There were no significant association with SES. Learning disorders were reported in mothers (*n* = 2), one father (*n* = 1), and siblings (*n* = 2). Language disorder was reported in one sister.

### Language assessment

Oral language was assessed by speech therapists using the Nouvelles Épreuves pour l'Examen du Langage battery.^19^ Children completed the full test battery. Nine subtests were selected based on their relevance to the domains of interest in this study: phonology, lexicon, and syntax, in both production and comprehension modes (Table 2 and Appendix S1). Raw scores were converted into age 7 years standardized z‐scores.

**TABLE 2:** Language variables.

Task name interest	Label	Language module	Modality
Phonological naming task, 1‐syllable words	Phono1	Phonology	Production
Phonological naming task, more than 1‐syllable words	Phono2		
Lexical naming task – concrete vocabulary	LexProd1	Lexicon	Production
Lexical naming task – abstract vocabulary	LexProd2		
Lexical comprehension task – concrete vocabulary	LexComp1	Lexicon	Comprehension
Lexical comprehension task – abstract vocabulary	LexComp2		
Morphosyntactic sentence completion task	SyntProd	Syntax	Production
Morphosyntactic comprehension task – list A	SyntComp1	Syntax	Comprehension
Morphosyntactic comprehension task – list B	SyntComp2		

Data were missing for four tasks in three children because of administration errors or participant fatigue.

### Statistical analysis

In this prospective longitudinal cohort study, analyses were performed with RStudio 2024.12.0 + 467 (Posit PBC, Boston, MA, USA). Significance was set at *p* < 0.05.

Principal component analysis with oblique rotation was used to identify the underlying dimensions in language performance and to reduce data dimensionality.^20^, ^21^ A hierarchical ascending classification with *k*‐means consolidation^22^ was applied to identify distinct subgroups based on composite language scores. Clusters were compared to age‐matched Nouvelles Épreuves pour l'Examen du Langage normative data^19^ using a one‐sample *t*‐test or a Wilcoxon signed‐rank test, depending on the distribution. Z‐score distributions across tasks categorized language proficiency as average (+1 to −1), borderline (−1 to −1.5), low (−1.5 to −2), or very low (less than −2). In the absence of specific cut‐offs provided by the authors of the Nouvelles Épreuves pour l'Examen du Langage test, we relied on thresholds commonly used in clinical practice.^23^ Differences in language outcomes across clusters were assessed using analysis of variance with a Tukey's post hoc test or a Kruskal–Wallis test with Dunn's post hoc test.

Demographic, environmental, and clinical variables were then compared across clusters using analysis of variance or a Fisher's exact test. To refine group comparisons, we compared cluster 1 (typically performing) against the other three clusters combined, using *t*‐tests or *χ* ^2^/Fisher's exact tests.

Variables identified as key factors of language outcomes were included in multiple linear regression models. Regression diagnostic addressed multicollinearity (variance inflation factors), distribution of residuals, residual independence (Durbin–Watson statistic), homoscedasticity (Breusch–Pagan test), and linearity, thus ensuring model validity.

Post hoc sensitivity analyses using balanced bootstrap resampling were performed to evaluate whether imbalances in lesion groups and arterial territory involvement could affect our results regarding the association between lesion characteristics and language outcomes. Across 1000 iterations, language outcomes were compared using a Wilcoxon rank‐sum test. Mean *p*‐values, percentages of significant iterations, and effect sizes were calculated.

## RESULTS

### Global distribution of language variables

Outliers were detected using visualizations and an interquartile range analysis, revealing three individuals with extremely low language scores, despite similar associated factors. Their inclusion did not alter the results of the analyses that followed, except for hierarchical clustering where it formed a separate cluster, thereby reducing the differentiation among the other subgroups. To optimize subgroup distinction and enhance interpretability, these participants were excluded, yielding a final sample size of 67 children whose language outcomes are reported in the results.

Principal component analysis with oblique rotation identified three components accounting for 73% of the total variance. The first component (30%) was related to lexical comprehension and production, the second (24%) to syntactic comprehension and production, and the third (20%) to phonological processing. These components were used to create three data‐driven composite language scores for each participant: lexicon composite score, syntactic composite score, and phonological composite score respectively. The detailed procedure and results are provided in Appendix S2.

### Four language profiles

**FIGURE 1:**
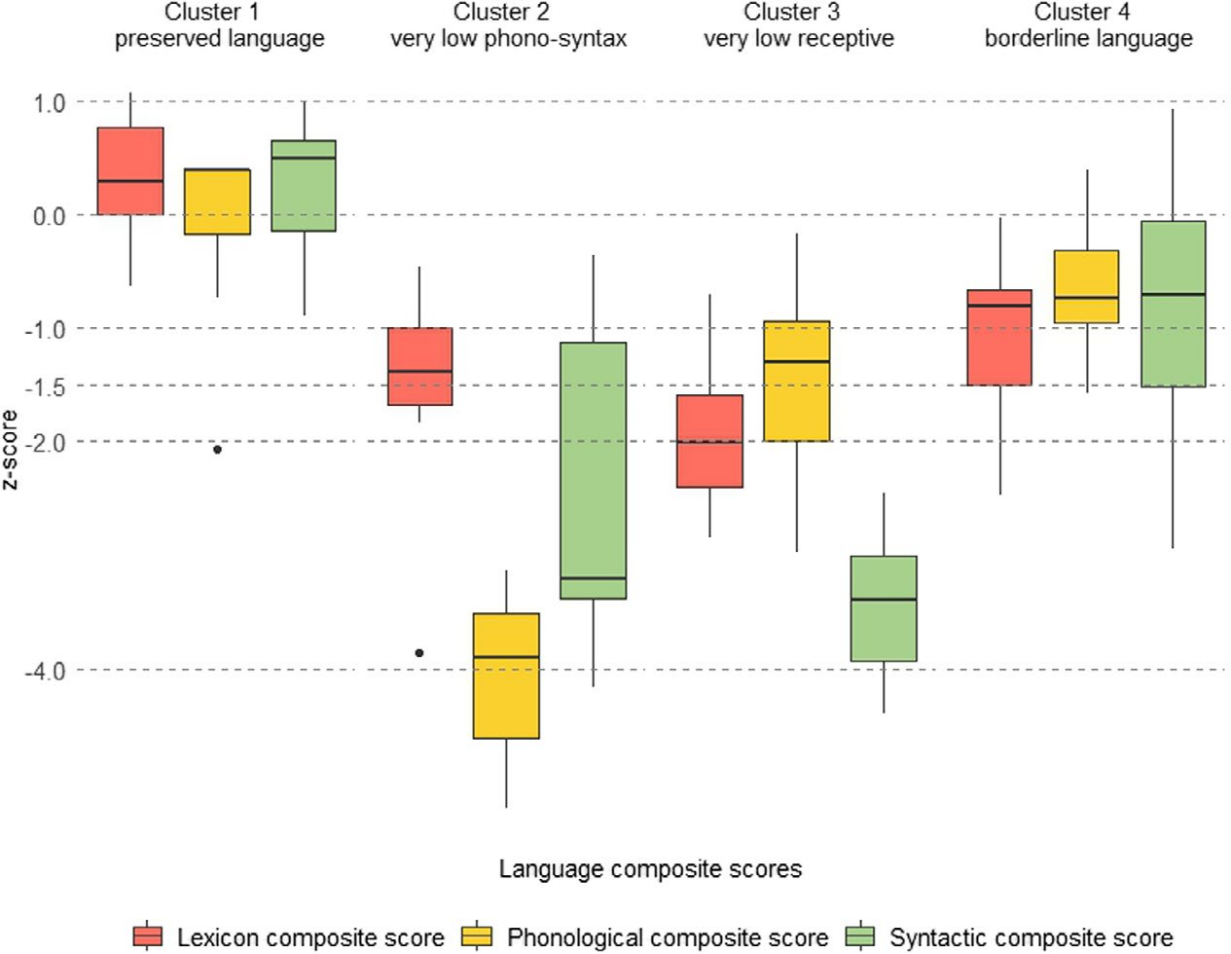
Distribution of language composite scores according to clusters. The dashed horizontal lines mark the median, the bottom and top boxes represent the 25th and 75th centiles, and the bottom and top vertical mark indicates the minimal and maximal values. The dots indicate extreme values.

Hierarchical ascending classification, followed by *k*‐means consolidation, identified four distinct clusters, each characterized by unique linguistic profiles (Figure 1). Further comparison of the nine initial language variables in participants from each cluster with normative data provided additional insights into the different profiles (Table 3). Cluster 1 (preserved language, *n* = 32): children performed within or above normative ranges across all tasks; cluster 2 (very low phono‐syntax, *n* = 7): children showed severe deficits in phonology and syntax; lexical comprehension was relatively preserved; cluster 3 (very low receptive, *n* = 11): children showed broad impairments, especially in receptive tasks, with borderline‐to‐low phonological production; cluster 4 (borderline language, *n* = 17): children had borderline scores in the lexical and syntactic production tasks, and in the syntactic comprehension task.

**TABLE 3:** Comparison of language score means for each cluster with age‐matched normative data.

Initial language scores	Normative sample		Cluster 1 Preserved language (*n* = 32)				Cluster 2 Very low phono‐syntax (*n* = 7)				Cluster 3 Very low receptive (*n* = 11)							Cluster 4 Borderline language (*n* = 17)				
	*n*	Mean (SD)	*n*	Mean	z‐score	*p*	Cohen's d	*n*	Mean	z‐score	*p*	Effect size	*n*	Mean	z‐score	*p*	Effect size	*n*	Mean	z‐score	*p*	Effect size
Phono1	108	27.26 (1.68)	32	27.00	−0.15	0.306	−0.18	7	19.86	−4.40	**0.022** *	−3.35	11	24.7	−1.78	**0.004** **	−3.35	17	25.77	−0.89	**0.006** **	−1.2
Phono2	108	49.13 (2.50)	32	49.53	0.16	0.078	0.32	7	39.71	−3.77	**0.022** *	−3.35	11	46.18	−1.18	**0.044** *	−2.15	17	48.29	−0.34	**0.050** *	0.84
Lex Comp1	108	35.20 (1.18)	32	*35.62*	0.36	***0.005*** *	0.54	7	34.43	−0.65	0.443	−2.27	11	32.27	−2.48	**0.005** **	−3.23	16	34.44	−0.64	0.217	1.91
Lex Comp2	108	20.08 (1.15)	32	20.38	0.26	0.054	0.35	7	18.14	−1.69	0.052	−3.11	11	17.64	−2.12	**0.004** **	−3.35	16	19	−0.94	**0.001** ***	−2.63
Lex Prod1	108	56.01 (8.54)	32	56.09	0.01	0.943	0.01	6	40.5	−1.82	**0.036** *	−3.35	11	40	−1.87	**0.004** **	−3.35	17	44.47	−1.35	**< 0.001** ***	−2.99
Lex Prod2	108	35.06 (3.90)	32	*37.56*	0.64	***< 0.001*** ***	0.82	7	27.57	−1.92	**0.023** *	−3.35	11	29.09	−1.53	**0.005** **	−3.23	17	31.53	−0.91	**0.002** **	−2.03
Synt Comp1	107	6.83 (1.06)	32	*7.22*	0.37	***0.037*** *	0.39	7	4.71	−2.00	**0.033** *	−3.23	11	2.82	−3.78	**0.004** **	−3.35	17	6.47	−0.34	0.667	4.66
Synt Comp2	104	7.38 (0.95)	32	*7.66*	0.29	***0.007*** **	0.51	7	4.57	−2.96	**0.022** *	−3.35	11	3.64	−3.94	**0.004** **	−3.35	17	6.41	−1.02	0.103	1.67
SyntProd	108	19.38 (6.66)	32	19.88	0.08	0.645	0.08	7	4.43	−2.24	**0.022** *	−3.35	10	6.1	−1.99	**0.006** **	−3.35	17	10.29	−1.36	**0.003** **	−1.67

A one‐sample *t*‐test or Wilcoxon signed‐rank test was used based on the data characteristics of each cluster. Bold type indicates statistical significance.

^*^ *p* ≤ 0.05,

^**^ *p* ≤ 0.01,

^***^ *p* ≤ 0.001. Italicized *p*‐values indicate a significantly higher score compared to normative data.

When compared to normative data, children in clusters 2, 3, and 4 demonstrated significantly lower results, with notable variability in their profiles (Table 3 and Figure 1).

Overall, considering the entire initial cohort of 72 children, including the two unable to complete the assessments (severe impairments), and the three outliers (extremely low scores), 40 children (56%) exhibited language difficulties at age 7 years.

### Factors associated with language outcomes

Analysis of non‐linguistic characteristics across clusters revealed several discriminating factors (Table 4). Bilingualism was more prevalent in the three clusters with borderline‐to‐low language skills. A family history of language or learning disabilities was notably frequent in the very low phono‐syntax group. Epilepsy was predominantly present in the clusters with the more severe language impairments (very low phono‐syntax and very low receptive groups), and was nearly absent in the preserved language group. FSIQ was within the normal range for the preserved language group, whereas it correlated with language severity in the other three clusters. When comparing children with borderline‐to‐very‐low language skills to those with fully preserved language abilities (Table 5), exposure to bilingualism (*p* = 0.014), epilepsy (*p* = 0.006), and FSIQ (*p* < 0.001), were confirmed as key discriminating variables. A family history of language or learning disorders did not significantly differentiate individuals between these two groups (*p* = 0.202), but it influenced variability within impaired profiles (Table 4).

**TABLE 4:** Comparison of descriptive data across clusters.

Characteristic	Preserved language (*n* = 32)	Very low phono‐syntax (*n* = 7)	Very low receptive (*n* = 11)	Borderline language (*n* = 17)	*p*
Sex					0.771
Female (*n* = 25)	14/32 (43.8)	2/7 (28.6)	4/11 (36.4)	5/17 (29.4)	
Male (*n* = 42)	18/32 (56.3)	5/7 (71.4)	7/11 (63.6)	12/17 (70.6)	
Birth order					0.093
First (*n* = 37)	19/32 (59.4)	3/7 (42.9)	9/11 (81.8)	6/17 (35.3)	
Not first (*n* = 30)	13/32 (40.6)	4/7 (57.1)	2/11 (18.2)	11/17 (64.7)	
Socioeconomic status (*n* = 67), mean (SD)	37.9 (13.8)	28.9 (11.8)	31.4 (11.9)	35.5 (11.8)	0.268
Exposure to bilingualism					**0.023** *
No (*n* = 57)	31/32 (96.9)	6/7 (85.7)	8/11 (72.7)	12/17 (70.6)	
Yes (*n* = 10)	1/32 (3.1)	1/7 (14.3)	3/11 (27.3)	5/17 (29.4)	
Family history of language or learning disabilities					**0.032** *
No (*n* = 60)	30/31 (93.8)	4/7 (57.1)	10/11 (90.9)	16/17 (94.1)	
Yes (*n* = 6)	1/31 (3.1)	3/7 (42.9)	1/11 (9.1)	1/17 (5.9)	
Epilepsy					**0.008** **
No (*n* = 53)	30/32 (93.8)	4/7 (57.1)	6/11 (54.5)	13/17 (76.5)	
Yes (*n* = 14)	2/32 (6.3)	3/7 (42.9)	5/11 (45.5)	4/17 (23.5)	
NAIS side					0.918
Left (*n* = 44)	21/32 (65.6)	5/7 (71.4)	7/11 (63.6)	11/17 (64.7)	
Right (*n* = 18)	8/32 (25.0)	1/7 (14.3)	4/11 (36.4)	5/17 (29.4)	
Bilateral (*n* = 5)	3/32 (9.4)	1/7 (14.3)	0/11 (0)	1/17 (5.9)	
Injury involving middle cerebral artery					0.678
No (*n* = 10)	4/32 (12.5)	1/7 (14.3)	3/11 (27.3)	2/17 (11.8)	
Yes (*n* = 57)	28/32 (87.5)	6/7 (85.7)	8/11 (72.7)	15/17 (88.2)	
NAIS location					0.488
Superficial (*n* = 47)	25/32 (78.1)	5/7 (71.4)	8/11 (72.7)	9/17 (52.9)	
Deep (*n* = 7)	2/32 (6.3)	0/7 (0)	1/11 (9.1)	4/17 (23.5)	
Both (*n* = 13)	5/32 (15.6)	2/7 (28.6)	2/11 (18.2)	4/17 (23.5)	
Cerebral palsy					0.501
No (*n* = 48)	25/32 (78.1)	5/7 (71.4)	6/11 (54.5)	13/17 (76.5)	
Yes (*n* = 19)	7/32 (21.9)	2/7 (28.6)	5/11 (45.5)	4/17 (23.5)	
Full‐scale IQ (*n* = 63), mean (SD)	106 (10.9)	74.0 (19.1)	76.4 (16.7)	92.7 (14.5)	**< 0.001** ***
Perceptual Reasoning Index (*n* = 66), mean (SD)	105 (11.8)	78.1 (14.7)	79.5 (17.7)	91.6 (13.6)	**< 0.001** ***
Schooling					**< 0.001** ***
Ordinary (*n* = 45)	29/32 (90.6)	1/7 (14.3)	3/11 (27.3)	12/17 (70.6)	
Ordinary with support (*n* = 18)	3/32 (9.4)	5/7 (71.4)	6/11 (54.5)	4/17 (23.5)	
Specialized (*n* = 4)	0/32 (0)	1/7 (14.3)	2/11 (18.2)	1/17 (5.9)	
Speech and language therapy					**< 0.001** ***
No (*n* = 48)	32/32 (100)	0/7 (0)	7/11 (63.6)	9/17 (52.9)	
Yes (*n* = 19)	0/32 (0)	7/7 (100)	4/11 (36.4)	8/17 (47.1)	

Data are *n* (%) unless stated otherwise. Calculated using an analysis of variance for quantitative variables and a Fisher's test for qualitative variables. Bold type indicates statistical significance.

Abbreviation: NAIS, neonatal arterial ischaemic stroke.

* *p* ≤ 0.05,

** *p* ≤ 0.01,

*** *p* ≤ 0.001.

**TABLE 5:** Comparison of descriptive data between the preserved language cluster and the other language profiles.

Characteristic	Preserved language (*n* = 32)	Borderline‐to‐very‐low language skills (*n* = 35)	*p*
Sex			0.43
Female (*n* = 25)	14/32 (43.8)	11/35 (31.4)	
Male (*n* = 42)	18/32 (56.3)	24/35 (68.6)	
Birth order			0.684
First (*n* = 37)	19/32 (59.4)	17/35 (46.8)	
Not first (*n* = 30)	13/32 (40.6)	18/35 (51.4)	
Socioeconomic status (*n* = 67), mean (SD)	37.9 (13.8)	32.9 (11.8)	0.119
Exposure to bilingualism			**0.014** *
No (*n* = 57)	31/32 (96.9)	26/35 (74.3)	
Yes (*n* = 10)	1/32 (3.1)	9/35 (25.7)	
Family history of language or learning disabilities			0.202
No (*n* = 60)	30/31 (93.8)	30/35 (85.7)	
Yes (*n* = 6)	1/31 (3.1)	5/35 (14.3)	
Epilepsy			**0.006** **
No (*n* = 53)	30/32 (93.8)	23/35 (65.7)	
Yes (*n* = 14)	2/32 (6.3)	12/35 (34.3)	
NAIS side			0.858
Left (*n* = 44)	21/32 (65.6)	23/35 (65.7)	
Right (*n* = 18)	8/32 (25.0)	10/35 (28.6)	
Bilateral (*n* = 5)	3/32 (9.4)	2/35 (5.7)	
Injury involving the middle cerebral artery			0.736
No (*n* = 10)	4/32 (12.5)	6/35 (17.1)	
Yes (*n* = 57)	28/32 (87.5)	29/35 (82.9)	
NAIS location			0.405
Superficial (*n* = 47)	25/32 (78.1)	22/35 (62.9)	
Deep (*n* = 7)	2/32 (6.3)	5/35 (14.3)	
Both (*n* = 13)	5/32 (15.6)	8/35 (22.9)	
Cerebral palsy			0.545
No (*n* = 48)	25/32 (78.1)	24/35 (68.6)	
Yes (*n* = 19)	7/32 (21.9)	11/35 (31.4)	
FSIQ (*n* = 63), mean (SD)	106 (10.9)	84.7 (17.7)	**< 0.001** ***
Perceptual Reasoning Index (*n* = 66), mean (SD)	105 (11.8)	85.3 (16.0)	**< 0.001** ***
Schooling			**< 0.001** ***
Ordinary (*n* = 45)	29/32 (90.6)	16/35 (45.7)	
Ordinary with support (*n* = 18)	3/32 (9.4)	15/35 (42.9)	
Specialized (*n* = 4)	0 (0)	4 (11.4)	
Speech and language therapy			**< 0.001** ***
No (*n* = 48)	32/32 (100)	16/35 (45.7)	
Yes (*n* = 19)	0/32 (0)	19/35 (54.3)	

Data are shown as *n* (%) unless stated otherwise. Calculated using an analysis of variance for quantitative variables and a Fisher's test for qualitative variables. Bold type indicates statistical significance.

* *p* ≤ 0.05,

** *p* ≤ 0.01,

*** *p* ≤ 0.001.

As these four variables may have crucial roles in shaping the linguistic profiles observed across clusters, multiple linear regression analyses quantified their contribution on the composite language scores (Table 6). Regression diagnostics tests confirmed model validity. The lexicon composite score increased significantly with higher FSIQ (*p* < 0.001), absence of bilingualism (*p* = 0.004), and absence of family history (*p* = 0.016), with an adjusted *R* ^2^ of 0.52. The phonological composite score was only predicted by family history (*p* < 0.001), whose absence was associated with better performance in this model, with an adjusted *R* ^2^ of 0.41. The syntactic composite score increased significantly with higher FSIQ (*p* = 0.007), with an adjusted *R* ^2^ of 0.36. After removing the FSIQ (because of its strong association with epilepsy, *p* < 0.001), epilepsy, exposure to bilingualism, and family history remained significant predictors of language outcomes. All three predicted the lexicon (adjusted *R* ^2^ = 0.38) and the phonological composite score (adjusted *R* ^2^ = 0.28). For syntax, only epilepsy and family history were significant (adjusted *R* ^2^ = 0.20). To reduce the impact of verbal components and motor speed constraints, we repeated the analyses on the Perceptual Reasoning Index. FSIQ and Perceptual Reasoning Index were strongly correlated (*r* = 0.86). Analyses yielded results highly consistent with those obtained using the FSIQ (Appendix S3).

**TABLE 6:** Determinants of domain‐specific language abilities.

Dependent variable	Explanatory variables	*β* (95% CI)	*p*	Adjusted *R* ^2^	Model *p*
Lexicon composite score	Epilepsy	−0.45 (−1.12 to 0.23)	0.189	0.52	**< 0.001** ***
	Family history	−0.93 (−1.68 to 0.18)	**0.016** *		
	FSIQ	0.03 (0.01–0.04)	**< 0.001** ***		
	Exposure to bilingualism	−0.86 (−1.44 to 0.28)	**0.004** **		
Phonological composite score	Epilepsy	−0.47 (−1.34 to 0.41)	0.288	0.41	**< 0.001** ***
	Family history	−2.07 (−3.04 to 1.10)	**< 0.001** ***		
	FSIQ	0.02 (0–0.04)	0.075		
	Exposure to bilingualism	−0.74 (−1.49 to 0.01)	0.054		
Syntactic composite score	Epilepsy	−0.71 (−1.77 to 0.35)	0.185	0.36	**< 0.001** ***
	Family history	−0.88 (−2.06 to 0.30)	0.140		
	FSIQ	0.03 (0.01–0.06)	**0.007** **		
	Exposure to bilingualism	−0.70 (−1.62 to 0.21)	0.129		

*n* = 62 (six observations were excluded because of missing data). *β*, unstandardized regression coefficient (estimate). 95% confidence intervals (CIs) are provided for each estimate. Bold type indicates statistical significance.

^*^ *p* ≤ 0.05,

^**^ *p* ≤ 0.01,

^***^ *p* ≤ 0.001. Abbreviation: FSIQ, full‐scale IQ.

### Schooling impact and speech and language intervention

Results revealed significant differences in schooling experiences among children across the various clusters (Table 4). As expected, children in the preserved language group largely attended mainstream schools without support. A similar trend was observed in the borderline language group. In contrast, most children in the very low phono‐syntax and very low receptive groups attended mainstream schools with support, indicating a greater level of functional challenges that require individualized educational interventions. A minority (6%) needed specialized schools.

Access to speech and language therapy varied across groups (Table 4). All children in the very low phono‐syntax group received intervention, whereas half of those in the borderline language group and only a third of those in the very low receptive group had accessed such support. Complementary analyses suggested that access to therapy was associated with language scores, IQ, and schooling, but not with other factors, such as SES or lesion characteristics.

## DISCUSSION

This prospective longitudinal cohort study aimed to analyse oral language outcomes at age 7 years after NAIS. The findings revealed four distinct language profiles with 56% of children displaying language performance significantly below age expectations. Epilepsy, exposure to bilingualism, a family history of language or learning disorders, and lower intellectual functioning were strongly associated with poorer language outcomes. The specific impact of each factor varied across language domains. Additionally, language impairments were associated with greater educational support needs. Finally, access to speech and language therapy was determined more by the nature of the language difficulties than by their severity.

### Language profiles

Contrary to studies suggesting that early language delays after neonatal stroke resolve over time,^24^ our findings confirm persistent language difficulties at school age, with 56% of children exhibiting language below age expectations. However, all children had developed functional language by age 7 years and none presented with aphasia.

To better reflect clinical realities and move beyond arbitrary thresholds,^25^ we used a data‐driven classification approach informed by clinical relevance. This allowed us to identify four distinct language profiles: preserved abilities and three impaired subtypes showing distinct phonological, lexical, and syntactic vulnerabilities. These findings build on previous observations in the same cohort,^10^ offering a more nuanced understanding of language trajectories after NAIS and confirming the persistence of diverse profiles long after the initial stroke.^12^ This aligns with prior research highlighting the presence of specific language profiles after NAIS.^13^

### Determinants of language development

We hypothesized that lesion characteristics, especially hemispheric side, would influence language outcomes given the early specialization of the left hemisphere for language.^3^ However, in our cohort, language deficits occurred irrespective of lesion side, with no clear differences in profile. This supports the notion of efficient right hemisphere compensation after early left‐sided lesions.^4^, ^26^ Similarly, arterial territory involvement did not predict specific language profiles, contrasting with prior studies suggesting an increased risk of deficits after middle cerebral artery involvement.^14^ The post hoc sensitivity analyses (Appendix S4) showed that only the syntsuggactic production measure suggested a possible effect of lesion side, aligning with prior findings;^27^ however, the observed effect was weak and probably negligible. For arterial territory, syntactic comprehension measures indicated potential subtle differences between middle cerebral artery and non‐middle cerebral artery lesions. However, these observations were based on very small subgroups and lacked statistical robustness. Overall, neither lesion side nor arterial territory demonstrated a systematic influence on language outcomes in this cohort. Future prospective studies with larger, balanced groups will be essential to clarify whether the observed trends reflect true effects or random variation.

Consistent with the prior literature, epilepsy was negatively associated with language abilities.^15^, ^26^ Previous studies extensively documented the link between epilepsy and global intellectual impairment,^14^, ^28^ a finding that our study also supports. In our analysis, epilepsy was associated with poorer language outcomes only when the FSIQ was excluded from the regression models, suggesting that its influence may be indirect and potentially mediated through broader cognitive effects. As our definition of epilepsy included cases requiring prolonged antiseizure treatment, the potential cognitive effects of these medications should also be considered when interpreting these associations.

Although a higher proportion of females with preserved language abilities was observed in the group, and despite previously documented anatomical brain differences,^29^ no significant sex‐related effects on language development were observed, which is consistent with data from the general population.^2^

In our cohort, SES was not associated with school‐age language outcomes. This contrasts with studies reporting early SES effects in typically developing children and after early brain injury.^1^, ^16^, ^30^ Composite SES measures can dilute the effects driven by specific components, such as maternal education.^31^ Many prior studies focused on toddlers and preschoolers, whereas our outcomes were measured at school age, when early SES effects may be attenuated through schooling and intervention.^31^ Moreover, most prior research was conducted in high‐income, English‐speaking countries, where social, educational, and health care contexts differ markedly from France, where the effect of SES may consequently be more moderate. Finally, unmeasured mediators, such as language input, therapy access, and parental engagement, may have masked any indirect effects.^1^, ^16^, ^30^, ^32^

Exposure to bilingualism was negatively associated with lexical outcomes, contrasting with some recent studies suggesting neutral or even positive effects, particularly for later‐onset strokes.^33^ The nature and timing of bilingualism probably have critical roles. Future research should incorporate more detailed measures (e.g. age at exposure, proficiency) to clarify its impact after NAIS.

A family history of language or learning disabilities was a significant predictor of language outcomes, particularly in phonology and vocabulary. This supports findings from the general population^34^ and suggests that familial risk factors should be considered even in the context of early brain injury.

General cognitive functioning, as indexed using the FSIQ, was strongly associated with lexical and syntactic outcomes. This supports the well‐documented link between cognitive performance and language development in children with and without early brain lesions.^14^, ^26^ However, whether cognitive delay precedes language deficits or whether both share a common origin remains unresolved.^14^ The comparable results obtained with the non‐verbal Perceptual Reasoning Index suggest that our findings regarding the association between cognitive ability and language outcomes are robust to the choice of cognitive index.

Finally, we found no association between CP and language skills at age 7 years, although this does not preclude the co‐occurrence of disorders, as shown previously.^10^

### Impact on schooling and speech and language intervention

Most children attended mainstream schooling, but those with severe language difficulties required additional educational support or specialized schooling, highlighting the functional impact on children's educational experiences. This is consistent with previous research suggesting that severe language deficits may act as a barrier to full school achievement.^35^

Children with very low phono‐syntactic abilities were more likely to receive speech and language therapy, whereas those with less observable difficulties, such as milder impairments or predominantly receptive language deficits, were less frequently referred, regardless of severity. This suggests that overt, audible difficulties are more readily identified and acted upon, leading to disparities in intervention access that appear driven more by symptom perceptibility than by actual severity. Beyond clinical presentation, the literature shows that access to paediatric care is shaped by systemic and perceptual factors. International studies highlight the role of professional and parental perceptions, cultural and linguistic barriers, service availability, and socioeconomic inequalities.^36^ In France, limited and uneven distribution of speech therapists leads to long delays and, at times, treatment abandonment, while socioeconomic and cultural variables further affect access.^37^, ^38^ Referral pathways also matter, as general practitioners—the main prescribers—may lack knowledge of therapy modalities.^39^ Our data did not allow for a detailed exploration of these systemic influences. In our cohort, SES and other background variables were not associated with speech therapy access. Future research would benefit from a deeper exploration of factors influencing access to therapy. Despite these limitations, our results underline the specific risk of underestimating language difficulties after paediatric stroke. Recent studies showed that research often focuses on motor outcomes, sometimes excluding children without motor impairment, potentially overlooking cognitive or language difficulties.^14^ Moreover, language assessments are frequently based on global cognitive tools that fail to capture the full complexity of language functioning.^25^ These findings underscore the need for targeted, domain‐specific language assessments beyond global neuropsychological tests to accurately detect language impairments, including subtle but functionally significant ones.

### Limitations

Although the language battery used was considered a reference in the French‐speaking context of this study, it has limited published psychometric data and lacks global indices. Thus, we created our own composite scores using data‐driven dimensionality reduction methods. While this approach is methodologically rigorous, it weakens the interpretability of the composite scores and limits comparability with other studies. These techniques have not yet been applied to neurotypical children, limiting generalizability.

Two children were not included in the analyses because of severe global cognitive impairments that made them unable to complete the language assessments. Additionally, three children with extremely low language scores were excluded to improve the interpretability of the clustering analysis because these outliers disproportionately influenced cluster formation. These exclusions may have biased our findings towards children with milder language difficulties. Thus, our sample may not capture the full range of language outcomes after NAIS, limiting the generalizability of our results. Moreover, the relatively small sample size (*n* = 67) may further limit the stability and generalizability of the results of the principal component analysis.

The 7‐year evaluation did not include direct measures of communication nor social participation, restricting our ability to assess the real‐world impact and functional relevance of the identified language profiles. Future studies should include such measures to better capture how these profiles translate into everyday communicative functioning and participation.

Finally, recent studies debated the role of post‐stroke functional brain reorganization in children's language development.^13^, ^17^ As our cohort lacked such data at age 7 years because of a high failure rate of language functional magnetic resonance imaging, we cannot document the links between contralesional plasticity and language abilities. Future research should further clarify these mechanisms.

### Conclusion

To the best of our knowledge, this is the first study to provide a thorough examination of oral language development in a highly homogeneous cohort of school‐age children after NAIS, with a notable sample size, considering the low frequency of the condition and the length of the follow‐up. Our findings highlight the high prevalence of language difficulties at age 7 years, with over half of children in our cohort showing impairments. While severe deficits were rare and no child presented with aphasia, this data‐driven study reveals distinct language profiles shaped by biological, cognitive, and environmental factors, including children with subtle or less noticeable difficulties. These children often develop seemingly functional language, which may mask underlying challenges. Notably, access to speech and language therapy depended more on the perceptibility of deficits than on their severity. This raises concerns about under‐identification and under‐referral of children with receptive or more nuanced impairments. Our research shows that implementing systematic and comprehensive language assessments for children with NAIS is crucial, ideally during the preschool age, to ensure timely and equitable access to interventions. Future research should integrate functional and neurobiological measures to better understand developmental trajectories and optimize care for children with a history of neonatal stroke.

## Supporting information


**Appendix S1:** Examples of the Nouvelles Épreuves pour l'Examen du Langage phonological naming tasks and lexical naming tasks

**Appendix S2:** Composite language scores: methodology and results

**Appendix S3:** Regression analyses using the Perceptual Reasoning Index

**Appendix S4:** Post‐hoc analyses, influence of NAIS side, and arterial territory on language outcomes

## Data Availability

Data available on request from the authors.
