# Supplementary material for: Oral language profiles and associated factors in children after neonatal arterial ischaemic stroke
Source: Dev Med Child Neurol. 2025 Dec 30;68(8):1105–16. doi: 10.1111/dmcn.70132 (PMC13340624; doi:10.1111/dmcn.70132)
Supplement: Supplementary file 1 — Appendix S1: Examples of the Nouvelles Épreuves pour l'Examen du Langage phonological naming tasks and lexical naming tasks [file DMCN-68-1105-s001.docx]

**Appendix S1** – Examples of the NEEL phonological naming tasks and lexical naming tasks

NEEL - phonological naming tasks


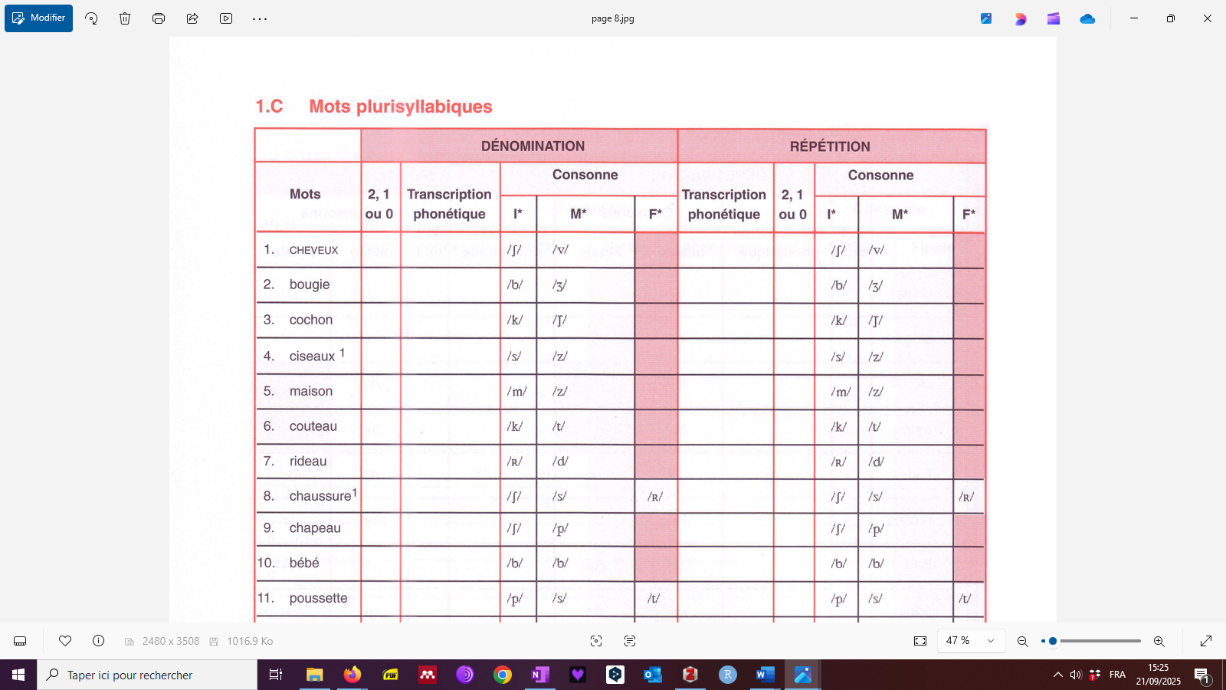

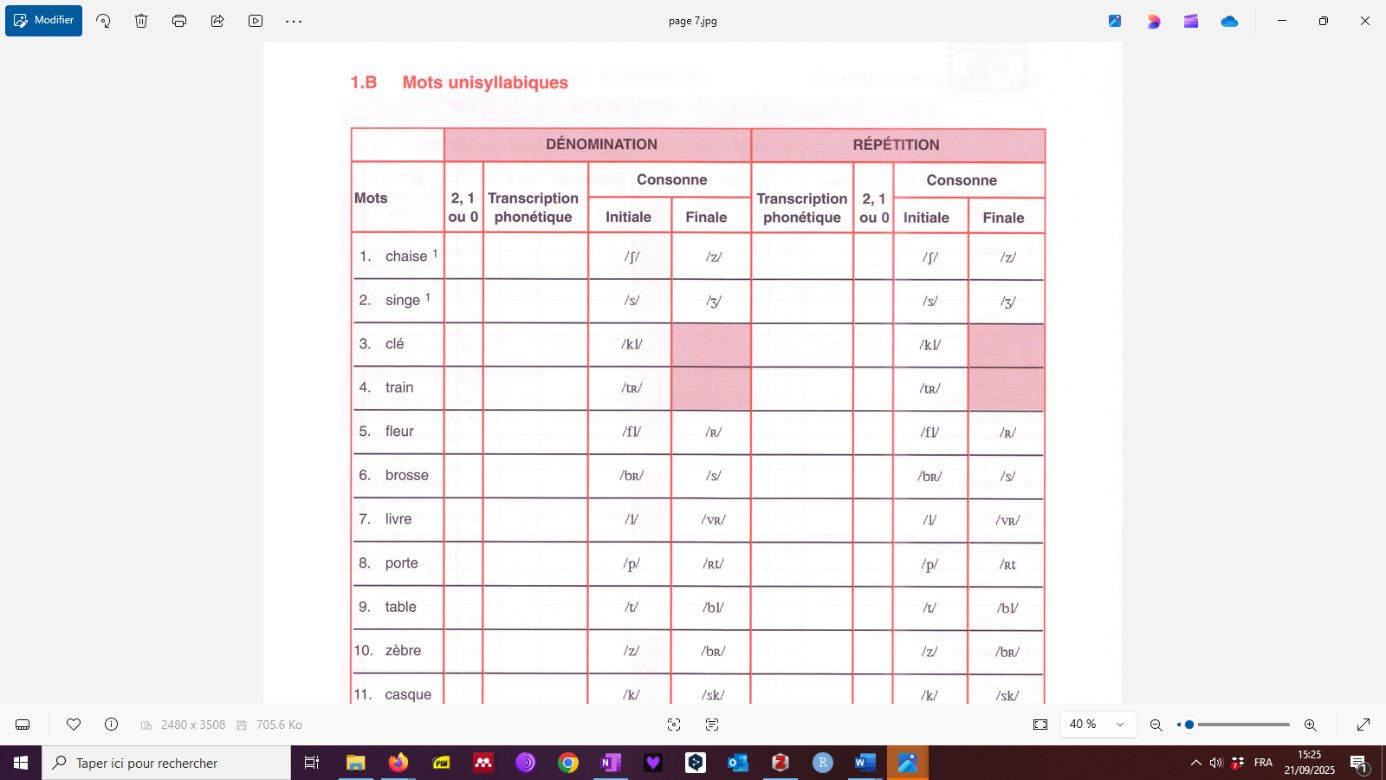
Children are asked to name images from two series differing in phonological complexity (monosyllabic vs. polysyllabic words). Full credit is given only if the target word is correctly produced with accurate phonology. No points are awarded if the response is incorrect or contains phonological errors (a specific scoring procedure is applied for persistent articulatory distortions (e.g., sigmatism)). In such cases, the examiner provides the correct word and asks the child to repeat it. Two scores are obtained: one for spontaneous production, and one for repetition.

NEEL - lexical naming tasks


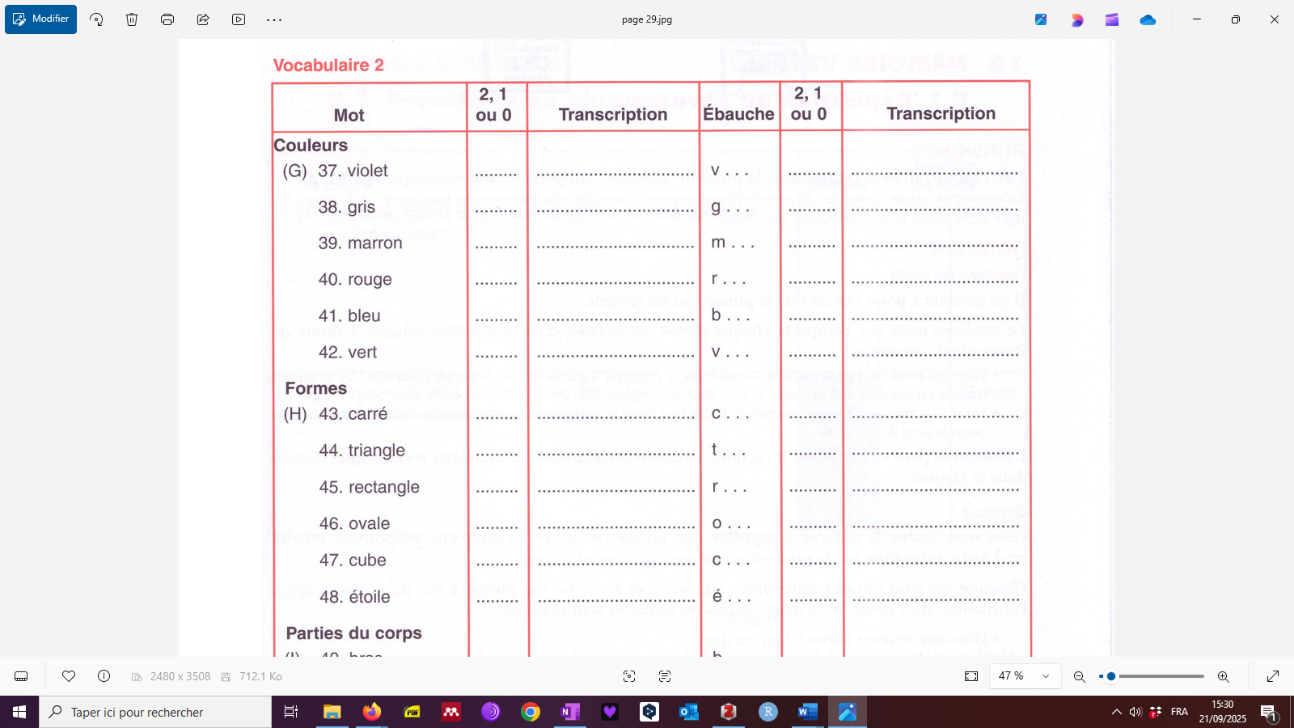

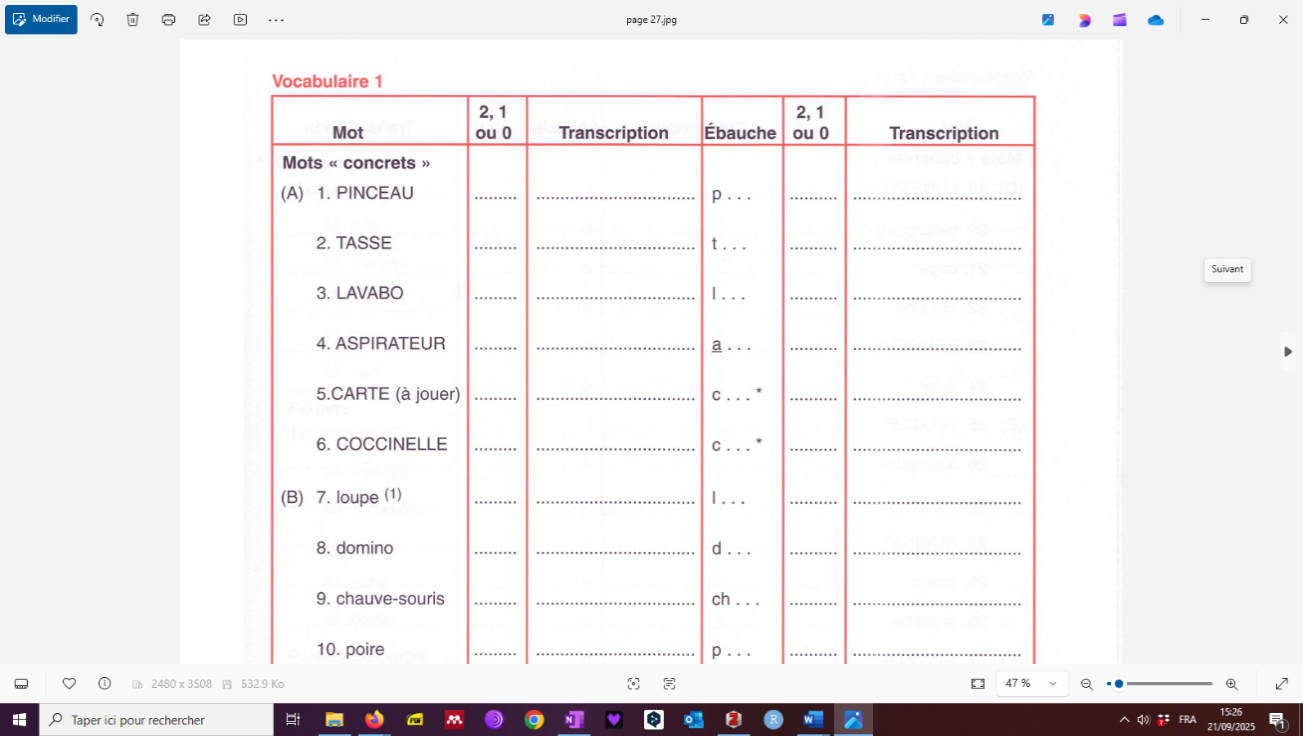
The lexical naming task also involves picture naming but focuses on lexical access. Here, points are given when the target word is produced, even if phonological errors are present. If the child fails to retrieve the word, an initial phonological cue is provided by the examiner. Two separate scores are again derived: one for spontaneous naming, and another after the cue is given.
