# Supplementary material for: Oral language profiles and associated factors in children after neonatal arterial ischaemic stroke
Source: Dev Med Child Neurol. 2025 Dec 30;68(8):1105–16. doi: 10.1111/dmcn.70132 (PMC13340624; doi:10.1111/dmcn.70132)
Supplement: Supplementary file 2 — Appendix S2: Composite language scores: methodology and results [file DMCN-68-1105-s002.docx]

**Appendix S2 –** Composite Language Scores: Methodology and Results

METHOD - Statistical analysis

Principal Component Analysis (PCA) was employed to identify the underlying dimensions of language performance, grouping highly correlated variables into main factors. Missing language scores were examined and addressed through multiple imputations. Considering the interdependent nature of language variables, an oblique rotation (Promax) was applied to relax the independence assumption, as suggested by Falissard^19^. By allowing the components to be correlated, the oblique rotation provides a more realistic representation of the data structure and enables a more accurate identification of the underlying dimensions. The number of factors retained was determined by testing several models and selecting the solution that provided the smallest number of factors while achieving a high proportion of explained variance (near 75%) and maintaining clinical interpretability.

These analyses led to the creation of data-driven composite scores, inspired by the work of Chao and Wu (2017)^20^. For each dimension identified by the PCA, only variables with loadings of 0.5 or higher were retained. The weight of each variable was calculated based on its loadings on the dimension using the following formula:

$$Weighed index of the variable V_{1}=\frac{| loading V_{1}|}{\sum|loading V_{1..i}|}$$

For each individual and each dimension, a composite score was computed by summing the individual z-scores of the retained variables, weighted by the previously calculated weighted index, as follows. These composite scores were used for further analysis.

$$Composite score=\sum(z-scores*weighted index)$$

RESULTS – Creating composite scores

**PCA results**

The first two axes of the PCA accounted for 64% of the total inertia of the dataset, which was significantly higher than the reference value given the dimensions of the dataset. This first plane therefore adequately represented the variability contained in a large portion of the data. The first factor was predominant, explaining 54.5% of the total data variability on its own, and it was the only axis that carried meaningful information.


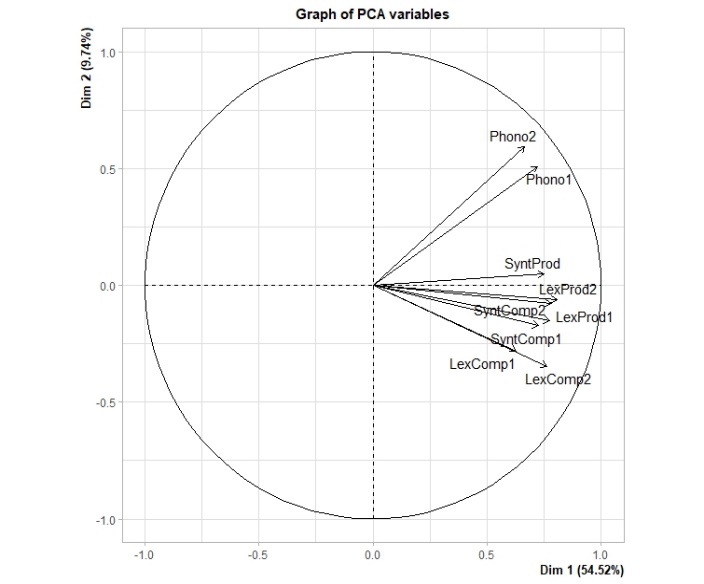

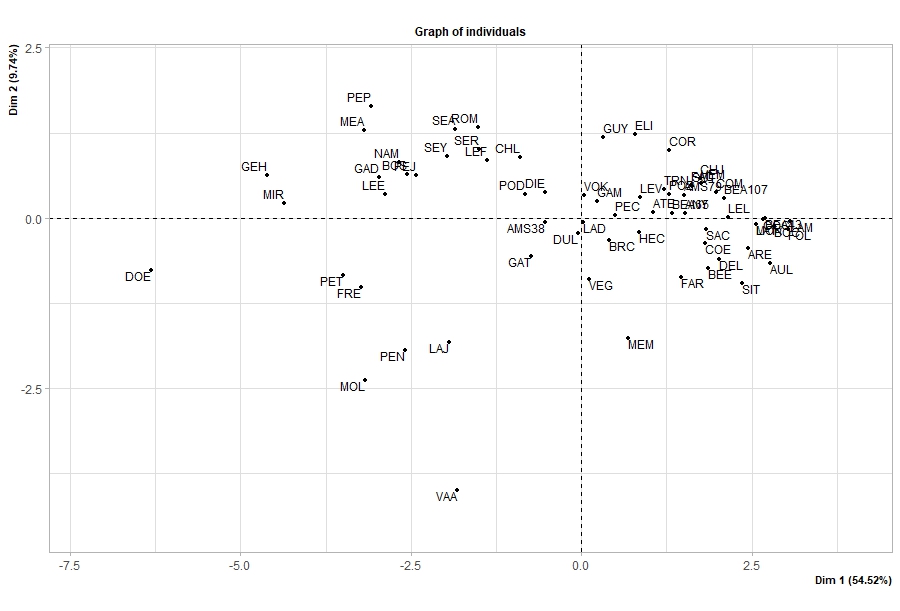


C Graph of individuals


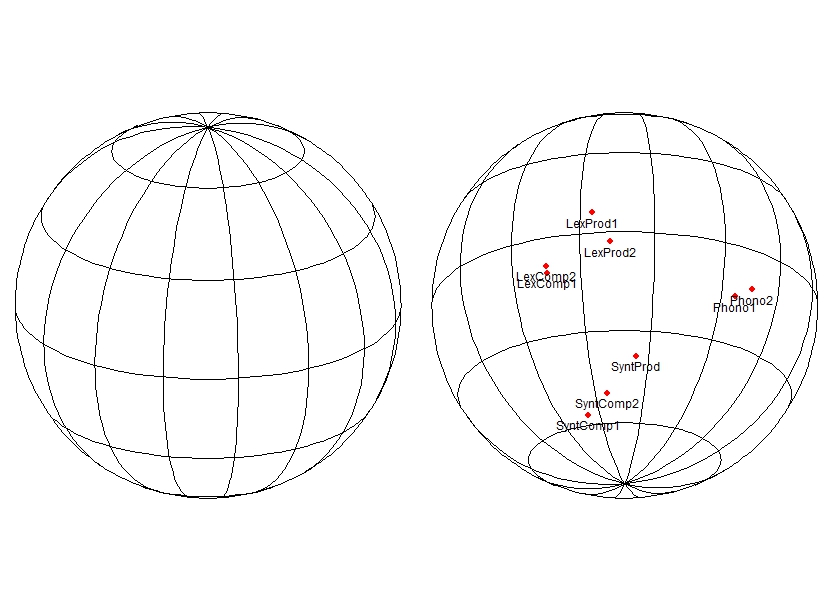


A Graph of PCA variables – 2 dimensions

B Graph of PCA variables - sphere

***Figure Supp 1.*** PCA results

The nine language variables contributed significantly to this dimension, with high and relatively homogeneous contribution from all variables. This dimension could be interpreted as a global language factor. While the predominance of a single factor is informative, it may obscure more subtle relationships between the variables.

**Standardized Factor Loadings and Complexity for Language Tasks (Oblique Rotation)**

The analysis with oblique rotation identified 3 components that together explained 73% of the total variance in the data. The first component (RC1) accounted for 30% of the variance and was primarily associated with lexical comprehension and production tasks. The second component (RC3), explaining 24% of the variance, was related to syntactic comprehension and production, while the third component (RC2) accounted for 20% of the variance and captured phonological processing. Most variables exhibited high communalities, indicating that the components effectively captured their variance, with minimal cross-loading.


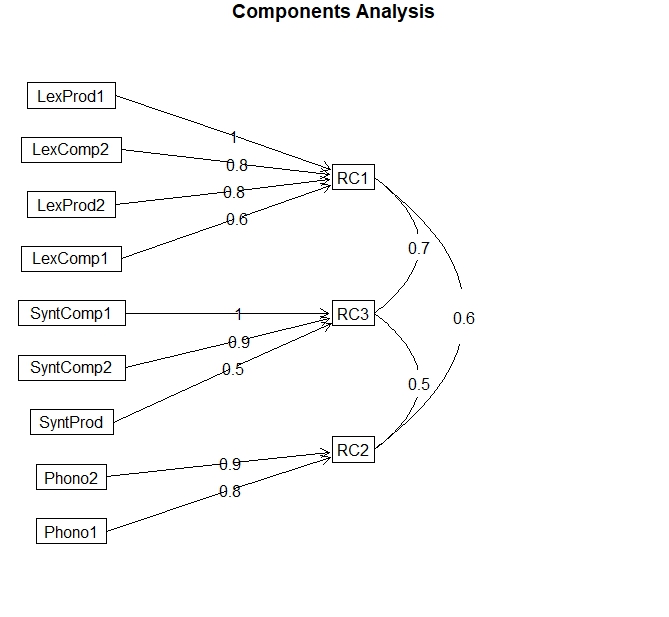


***Figure Supp - 2.*** Factorial analysis results after oblique rotation

The correlations between components suggested that these linguistic abilities were not entirely independent, justifying the use of oblique rotation.

The model demonstrated a good fit, with a low RMSR value (0.07) and a high off-diagonal fit index (0.98). A significant chi-square test (25.97, *p* < 0.011) further supported the adequacy of the three-component solution.

Based on these results, 3 composite scores were calculated for each participant in the NAIS group. To improve readability, the factors RC1, RC2 and RC3 were renamed "RC Lexicon", "RC Phono" and "RC Syntax" respectively in the further analyses.
