# Supplementary material for: Oral language profiles and associated factors in children after neonatal arterial ischaemic stroke
Source: Dev Med Child Neurol. 2025 Dec 30;68(8):1105–16. doi: 10.1111/dmcn.70132 (PMC13340624; doi:10.1111/dmcn.70132)
Supplement: Supplementary file 3 — Appendix S3: Regression analyses using the Perceptual Reasoning Index [file DMCN-68-1105-s003.docx]

**Appendix S3** - Regression analyses using the Perceptual Reasoning Index (PRI)

| Dependant variables | Explicative variables | β | *p*-value | Adjusted R^2^ | | F | *p*-value |
| --- | --- | --- | --- | --- | --- | --- | --- |
| Lexicon composite score | Epilepsy | -0.58 | 0.062 | 0.50 | (4.60) 15.17 | | **<0.001***** |
|  | Family history | -1.23 | **0.001*** |  |  |  |  |
|  | PRI | 0.02 | **0.004**** |  |  |  |  |
|  | Bilingualism exposure | -0.91 | **0.003**** |  |  |  |  |
| Phonologic composite score | Epilepsy | -0.39 | 0.372 | 0.36 | | (4.60) 8.62 | **<0.001***** |
|  | Family history | -2.09 | **<0.001***** |  |  |  |  |
|  | PRI | 0.02 | **0.046*** |  |  |  |  |
|  | Bilingualism exposure | -0.57 | 0.183 |  |  |  |  |
| Syntactic composite score | Epilepsy | -0.49 | 0.313 | 0.42 | | (4.60) 10.65 | **<0.001***** |
|  | Family history | -0.95 | 0.098 |  |  |  |  |
|  | PRI | 0.05 | **<0.001***** |  |  |  |  |
|  | Bilingualism exposure | -0.42 | 0.373 |  |  |  |  |
