# Supplementary material for: Oral language profiles and associated factors in children after neonatal arterial ischaemic stroke
Source: Dev Med Child Neurol. 2025 Dec 30;68(8):1105–16. doi: 10.1111/dmcn.70132 (PMC13340624; doi:10.1111/dmcn.70132)
Supplement: Supplementary file 4 — Appendix S4: Post‐hoc analyses, influence of NAIS side, and arterial territory on language outcomes [file DMCN-68-1105-s004.docx]

**Appendix S4** – Post-hoc sensitivity analyses: influence of NAIS side and arterial territory on language outcomes

Additional post-hoc sensitivity analyses using a balanced bootstrap resampling approach to evaluate whether the imbalance in lesion groups (44 left-sided, 18 right-sided, and 5 bilateral lesions) and arterial territory involvement (57 MCA vs. 10 non-MCA lesions) may have influenced the observed lack of association between lesion characteristics and language outcomes.

Given the small number of participants with bilateral lesions, and considering the low prevalence of bilateral neonatal stroke reported in the literature, we focused our analyses on comparing left- versus right-sided lesions.

A random subset of participants was drawn from the left-lesioned group to match the size of the right-lesioned group in 1000 iterations, and comparisons of language outcomes were performed using Wilcoxon tests. From these analyses, we calculated the mean p-value across all iterations, the percentage of iterations yielding significant results, and the corresponding effect size.

The results for the left versus right lesion comparison are summarized below:

| **Variable** | **Mean_p** | **Percent_signif** | **Mean_d** |
| --- | --- | --- | --- |
| **Phono1** | 0,421 | 11,6 | 1,036 |
| **Phono2** | 0,497 | 5,5 | 0,809 |
| **LexProd1** | 0,445 | 9,6 | 0,437 |
| **LexProd2** | 0,435 | 9,4 | 0,617 |
| **LexComp1** | 0,488 | 6,1 | 0,843 |
| **LexComp2** | 0,464 | 7,5 | 0,879 |
| **SyntProd** | 0,344 | 17,6 | 0,670 |
| **SyntComp1** | 0,420 | 11,4 | 1,031 |
| **SyntComp2** | 0,507 | 5,3 | 0,789 |
| **Lexicon comp score** | 0,413 | 11,2 | 0,613 |
| **Phono comp. score** | 0,498 | 5,1 | 0,801 |
| **Syntactic comp. score** | 0,425 | 11,3 | 1,001 |

The same balanced bootstrap analyses with 1000 iterations were also conducted for arterial territory involvement, comparing participants with middle cerebral artery (MCA) lesions (n = 57) versus those with non-MCA lesions (n = 10).

| **Variable** | **Mean_p** | **Percent_signif** | **Mean_d** |
| --- | --- | --- | --- |
| **Phono1** | 0,495 | 6,7 | 0,653 |
| **Phono2** | 0,499 | 4,4 | 0,806 |
| **LexProd1** | 0,419 | 11,3 | -0,075 |
| **LexProd2** | 0,482 | 7,9 | 0,176 |
| **LexComp1** | 0,475 | 6,4 | 0,869 |
| **LexComp2** | 0,485 | 5,3 | 0,633 |
| **SyntProd** | 0,507 | 5,7 | 0,612 |
| **SyntComp1** | 0,342 | 16,9 | 0,856 |
| **SyntComp2** | 0,302 | 24,5 | 1,402 |
| **Lexicon comp score** | 0,438 | 9,4 | 0,323 |
| **Phono comp. score** | 0,467 | 8,0 | 0,665 |
| **Syntactic comp. score** | 0,345 | 17,8 | 0,908 |
